# Dr. James Monroe Garvey

**Published:** 1909-03

**Authors:** 


Dr. James Monroe Garvey, of Tower, Mich., a graduate of the University of Buffalo, Medical Department, 1882, died in a sanitarium at Traverse City, Mich., February 1, 1909, aged 69 years.

